# Evaluation of Delirium Risk Factors in Intensive Care Patients

**DOI:** 10.4274/TJAR.2024.241526

**Published:** 2024-12-16

**Authors:** Selin Erel, Eda Macit Aydın, Bijen Nazlıel, Lale Karabıyık

**Affiliations:** 1 Gazi University Faculty of Medicine, Department of Anaesthesiology and Reanimation, Ankara, Turkey; 2 Gazi University Faculty of Medicine, Department of Anaesthesiology and Reanimation, Division of Intensive Care, Ankara, Turkey; 3 Gazi University Faculty of Medicine, Department of Neurology, Ankara, Turkey

**Keywords:** Cognition disorders, critical care, delirium, incidence, risk

## Abstract

**Objective:**

The negative effects of delirium in intensive care unit (ICU) patients necessitate the identification and management of risk factors. This study aimed to determine the incidence of delirium and its associated modifiable and non-modifiable factors in the ICU setting to provide valuable insights for better patient care and outcomes.

**Methods:**

Patients admitted to the ICU underwent delirium screening twice daily. Comprehensive records of modifiable and non-modifiable risk factors were maintained throughout the ICU stay.

**Results:**

The incidence of delirium was 32.5%. Age [odds ratio (OR) 1.04, confidence interval (CI) 1.02-1.06, *P* < 0.001)]. Illiteracy (OR 4, CI 1.19-13.35, *P*=0.02), hearing impairment (OR 3.37, CI 1.71-7.01, *P*=0.001), visual impairment (OR 3.90, CI 2.13-7.15, *P* < 0.001), hypertension (OR 2.56, CI 1.42-4.62, *P*=0.002), Sequential Organ Failure Assessment score (OR 1.21, CI 1.08-1.36, *P*=0.001), Acute Physiology and Chronic Health Evaluation II score (OR 1.20, CI 1.12-1.28, *P* < 0.001), presence of a nasogastric catheter/drain (OR 2.15, CI 1.18-3. 90, *P*=0.01), tracheal aspiration (OR 3.63, CI 1.91-6.90, *P* < 0.001), enteral nutrition (OR 2.54, CI 1.12-5.76, *P*=0.02), constipation (OR 1.65, Cl 1.11-2.45, *P*=0.02), oliguria (OR 1.56, Cl 1.06-2.28, *P*=0.02), midazolam infusion (OR 3. 4, Cl 1.16-10.05, *P*=0.02), propofol infusion (OR 2.91 Cl 1.03-8.19, *P*=0.04), albumin use (OR 2.39, Cl 1.11-5.14 *P*=0.02) and steroid use (OR 2.17, Cl 1.06-4.40, *P*=0.03) were found to be independent risk factors for delirium.

**Conclusion:**

This study highlights several risk factors contributing to delirium, such as age, sensory impairment, educational level, procedural interventions, and medications.Oral nutrition and mobilization are effective strategies for reducing delirium incidence in the ICU.

**Main Points**

• Delirium is prevalent within intensive care unit (ICU) environments, and the incidence of hypoactive type delirium is higher than expected.

• Various risk factors contribute to delirium, including age, sensory impairment, education level, procedural interventions, and drugs.

• Strategies such as oral nutrition and mobilization can help reduce delirium incidence in the ICU.

## Introduction

Delirium is a mental syndrome with a sudden onset in hospitalized patients, characterized by impaired cognitive function, and is more common in intensive care units (ICU).^1, 2^ This syndrome has many consequences, including prolonged hospital stays, elevated mortality rates, escalated healthcare costs, and a surge in the workload of healthcare providers.^3, 4^ Therefore, increasing the awareness of medical personnel regarding this syndrome in critically ill patients is of great importance for effective delirium management.^5^

The prevalence of delirium in ICUs is 22-84% and is higher and more variable than that in ward patients.^6, 7^ This variability may be due to the study design, differences in data collection, method of diagnosing delirium, patient characteristics, environmental conditions, and use of sedatives and analgesics. There are three classic types of delirium: hyperactive, hypoactive, and mixed. The diagnosis of hypoactive and mixed subtypes of delirium might be overlooked. However, these clinical subtypes may still be present in approximately one-third of all critically ill patients admitted to the ICU.^8, 9^

Delirium is a multifactorial syndrome. The risk factors are classified as modifiable or non-modifiable. Advanced age, comorbidities, and visual impairment are examples of non-modifiable factors. Electrolyte and metabolic abnormalities, drugs, infection, pain, anaesthesia and surgery, intensive care interventions, malnutrition, mobilization, and lack of environmental stimulation are among the most common modifiable factors.^10, 11^ During the coronavirus disease-2019 pandemic, delirium was observed more frequently due to the mandatory stricter implementation of isolation measures in ICUs. Benzodiazepine use and absence of family visits were modifiable risk factors for delirium in this patient group.^12^

Early recognition of delirium is important for all intensive care patients. Identifying risk factors associated with delirium and closely monitoring high-risk patients are crucial to ensure effective management and care. The primary approach to delirium prevention involves addressing and eliminating modifiable risk factors. However, in situations where delirium is accompanied by severe agitation, pharmacological treatment may be necessary to ensure patient safety and enhance comfort.^13, 14^ Although drugs such as antipsychotics, benzodiazepines, and dexmedetomidine have been used to treat delirium symptoms, current guidelines do not support their routine use.^11, 13, 14, 15, 16^

In this prospective observational study, we aimed to determine the overall incidence of delirium, including hypoactive and mixed types that may be clinically overlooked, and to identify the associated modifiable and non-modifiable risk factors in ICU patients.

## Methods

After obtaining ethics committee approval from the Gazi University Faculty of Medicine Clinical Research Ethics Committee (decision no.: 588, date: 10.9.2018), this prospective observational study was conducted between September 2018 and May 2019 at the Gazi University, Departments of Anaesthesiology ICU, Neurology, and General Surgery. All three tertiary ICUs involved in this study follow an arena-style design, which allows for optimal monitoring and patient care in a centralized setting.

**Figure 1:**
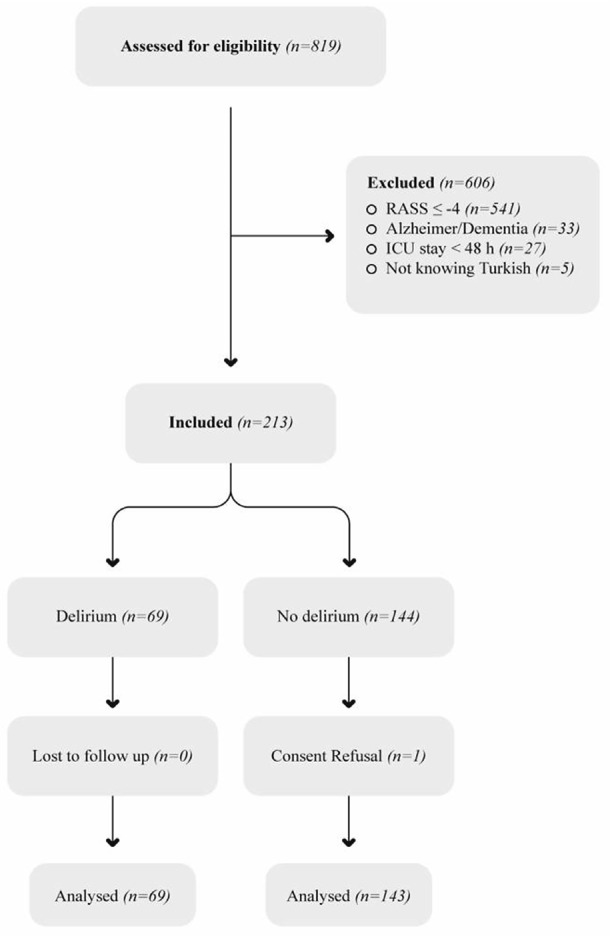
Flow chart of the study. RASS; Richmond agitation and sedation score; ICU, intensive care unit.

Patients aged >18 years of age who were hospitalized in the ICU for >48 hours were included in the study. Patients who lacked proficiency in Turkish, failed to comply with the diagnostic test (Richmond Agitation and Sedation Score: RASS ≤-4), or had a documented medical history of Alzheimer’s disease and/or dementia were excluded from the study (Figure 1).^17^ Diagnosis of Alzheimer’s disease, based on medical records before ICU admission. After ICU admission, patients with appropriate clinical conditions were screened using the Mini-mental test.

The evaluations were performed by the same anaesthesiologist, ensuring consistency across all patient assessments. This researcher was responsible for conducting twice-daily delirium screening tests at 8:00 am and 8:00 pm using the Confusion Assessment Method for the ICU to screen for delirium.^18^ Demographic data (age, gender, body mass index, education level, and comorbidities), ICU type (anaesthesia, neurology, and general surgery), ward type before ICU admission, duration of hospital stay before ICU admission, Acute Physiology and Chronic Health Evaluation (APACHE) II score, and Sequential Organ Failure Assessment (SOFA) score were recorded.

The daily follow-up records included the presence of medical devices (such as endotracheal tubes, nasogastric tubes, and catheters), duration of mechanical ventilation, drug infusions, pain scores [using the visual analogue scale (VAS)], hemodynamic changes, oxygen requirements, shock status, electrolyte and acid-base imbalances, number of administered drugs, family visits, method of nutrition, urine output, frequency of defecation, infection status, and administration of sedative drugs. Patients diagnosed with delirium were continuously monitored. Follow-up involved tracking the specific drug treatments, total duration of delirium, and delirium relapse. Additionally, the length of ICU stay and discharge or mortality status of the patients were recorded.

To ensure objective data collection, the records were maintained by an independent investigator who was not involved in patient follow-up or treatment. Routine patient care, including the management of delirium and other medical conditions, was provided by intensivist who were not a part of the study, ensuring that treatment protocols were unaffected by the research procedures.

### Statistical Analysis

The Statistical Package for the Social Sciences version 22.0 (SPSS Inc. Chicago, USA) was used to analyze the study data. The descriptive statistics section evaluates categorical variables as numbers and percentages and continuous variables as mean ± standard deviation. The conformity of continuous variables to a normal distribution was evaluated using visual (histogram and probability graphs) and analytical methods (Kolmogorov-Smirnov/Shapiro-Wilk tests). The Mann-Whitney U test was used to compare data that did not conform to the normal distribution. Pearson’s chi-square test was used to compare independent groups for categorical variables. Univariate logistic regression analysis was used to identify factors associated with delirium. The regression analysis results are presented as OR and 95% CI. The statistical significance level was set at *P *< 0.05.

## Results

In total, 212 patients from the anaesthesia, general surgery, and neurology ICUs were followed up. The most common indication for ICU admission was postoperative monitoring and care. An overview of admission indications categorized by ICU type is provided in Table 1.

The overall incidence of delirium in all ICUs was 32.5%. The mean age of the patients was 57.5 years, and 49.5% were male (Table 2). Among the patients diagnosed with delirium, 49.3% were hypoactive, 36.2% were hyperactive, and 14.5% were mixed-type. Delirium emerged on an average of 2.1±1.9 days and patients who experienced delirium remained in this state for an average of 5.8±6.4 days.

A significant difference was found between the patient groups when comparing the reasons for ICU admission and incidence of delirium (*P *< 0.001). Delirium occurred in 43.5% (n = 10) of patients admitted for respiratory failure, 50% (n = 2) of those with renal failure, 21.7% (n = 5) of trauma patients, 19.3% (n = 16) of postoperative patients, 64.3% (n = 9) of those with multiorgan dysfunction, 46.9% (n = 15) of patients with cerebrovascular incident, 75% (n = 3) of patients with sepsis, 4.3% (n = 1) of patients with intoxication, and 25% (n = 4) of those with acute abdomen.

Age (OR 1.04, CI 1.02-1.06, *P *< 0.001), illiteracy (OR 4, CI 1.19-13.35, *P*=0.024), hearing impairment (OR 3.37, CI 1. 71-7.01,* P*=0.001), visual impairment (OR 3.90, CI 2.13-7.15, *P *< 0.001), and hypertension (OR 2.56, CI 1.42-4.62, *P*=0.002) were found to be risk factors. Increased SOFA (OR 1.21, CI 1.08-1.36, *P*=0.001) and APACHE II (OR 1.20, CI 1.12-1.28, *P* < 0.001) scores at admission were found to be risk factors for delirium (Table 1).

Patients with delirium had a longer mean length of ICU stay (12.2±12.4 days) than those without delirium (6.4±5.5 days) (*P *< 0.001). The length of ICU stay was found to be a risk factor for delirium (OR 1.10, CI 1.05-1.15, *P* < 0.001). No significant correlation was observed between the number of days spent in the hospital before ICU admission and the development of delirium in the ICU.

The mortality rate was significantly higher in patients with delirium (24.6%) than in those without (4.9%) (*P *< 0.001) (Table 2).

During ICU stay, the presence of a nasogastric catheter and/or drain (OR 2.15, CI 1.18-3.90, *P*=0.012), tracheal aspiration (OR 3.63, CI 1.91-6.90, *P *< 0.001), and the frequency of aspiration (OR 1.35, CI 1.22-1.50, *P *< 0.001) were identified as risk factors for delirium. Patients with delirium were aspirated an average of 3.9±0.5 times a day, whereas those without delirium had an average of 0.6±0.1 aspirations per day. No significant association was found between the presence of a urinary catheter and the emergence of delirium (*P*=0.26) (Table 3).

In the comparison of feeding methods, enteral feeding was identified as a risk factor for delirium (OR 2.54, CI 1.12-5.76, *P*=0.025), whereas oral feeding was found to significantly decrease the incidence of delirium (OR 0.27, CI 0.13-0.55,* P*<0.001). However, no significant association was observed between parenteral nutrition and delirium emergence (*P*=0.81).

A defecation time of >3 days (OR 1.65, CI 1.11-2.45, *P*=0.02) and anuria/oliguria (OR 1.56, CI 1.06-2.28, *P*=0.02) were identified as risk factors for delirium.

Midazolam administration (OR 3.4, CI 1.16-10.05, *P*=0.02) and propofol infusion (OR 2.91, CI 1.03-8.19, *P*=0.04) were associated with an increased risk of delirium.

Albumin and steroid use was observed in 23.2% and 26.4% of patients with delirium, respectively. Albumin (OR 2.39, CI 1.11-5.14, *P*=0.02) and steroids (OR 2.17, CI 1.06-4.40, *P*=0.03) were found to be risk factors for delirium.

Hemodynamic instability was also identified as a risk factor for delirium, hypotension (OR 2.5, CI 1.01-6.26, *P*=0.04), and hypertension (OR 2.20, CI 1.11-4.74, *P*=0.021), increasing the risk of delirium.

On the other hand, mobilization was found to decrease the risk of delirium (OR 0.38, CI 0.20-0.73, *P*=0.003).

Irregular night sleep and sleep quality deterioration increase the risk of delirium. Among the patients with delirium, 56.5% described their night sleep as poor, 17.4% as moderate, and 26.1% as good (*P *< 0.001).

No statistically significant correlations were found between the frequency of family visits, dialysis, continuous renal replacement therapy, pain score, average number of daily medications taken, electrolyte levels, and delirium.

The mean VAS score was 1.97±0.29 in patients who developed delirium, compared to 2.26±0.18 in those who did not. No significant relationship was observed between pain scores and the development of delirium (*P*=0.16).

However, a significant relationship was observed between blood carbon dioxide level and delirium. Hypoxia (OR 2.05, CI 1.03-4.08, *P*=0.03) and hypercarbia (OR 2.0, CI 1.05-4.28, *P*=0.03) were identified as risk factors for delirium. No significant association was observed between hyperoxia and delirium in the present study.

Of the 69 patients with delirium, 43.5% received treatment for delirium. Pharmacological treatment was administered to 58.3% of patients in the anaesthesia, 41.7% in neurology, and 19.0% in general surgery ICUs. There was a statistically significant difference in the administration of pharmacological treatment for delirium between the different ICUs (*P*=0.015).

In 13 patients with delirium (39.4%), dexmedetomidine was used, while haloperidol was administered in 12 patients (36.4%), antipsychotics in 2 patients (6.1%), a combination of dexmedetomidine and haloperidol in 4 patients (12.1%), and a combination of haloperidol and antipsychotics in 2 patients (6.1%). No significant differences were observed between these pharmacological treatments in terms of the success of delirium management or mortality (*P*=0.8 and *P*=0.7, respectively). The treatments were continued for an average of 3.7±3.3 days during delirium management.

## Discussion

In the present study, the incidence of delirium was 32.5% among the 212 patients. The incidence of delirium, a multifactorial syndrome, varies due to various contributing factors. These variations can be attributed to differences in demographic profiles, varying levels of illness severity, distinct ICU features, and the utilization of diverse delirium screening tests in intensive care patients.

The type of ICU setting significantly affects the incidence, risk factors, and prognosis of delirium. The study was conducted across three different ICUs, each serving different patient populations. However, this variability may have positively influenced the results and increased the generalizability of the study. The prevalence of delirium may be higher in branch ICUs. For example, in one study, the incidence of delirium in non-intubated intensive care unit patients was only 20%, whereas in another study, this rate was found to be 83% in patients on mechanical ventilation.^18, 19^ In a comprehensive meta-analysis of 42 studies that focused on delirium in ICU patients, the overall incidence was 31.8%.^2^ Remarkably, the incidence in our study is consistent with that reported in the literature.

According to the results of our study, advanced age, visual and hearing impairments, hypertension and heart disease, illiteracy, and high APACHE II and SOFA scores during hospitalization were found to be the “non-modifiable risk factors” for delirium. Nasogastric catheter, wound drain, enteral nutrition, mechanical ventilation, oxygen requirement, midazolam and propofol infusion, albumin, and steroid use, decreased urine output and defecation frequency, hemodynamic instability, and infection were “modifiable risk factors” for the development of delirium (Table 3).

Despite being frequently overlooked in ICU patients, delirium has a significant impact on outcomes, increased length of ICU stay, and increased mortality rates.^2, 3, 10^ In our study, we found that patients who did not experience delirium had an average ICU stay of 6.4 days. On the other hand, those who developed delirium were hospitalized for a much longer period of 12.2 days, and mortality was observed to be higher in these patients. Various studies have also shown that delirium can lead to mortality rates ranging from 25% to 33%, even in ward patients, and can increase mortality in the intensive care unit by 1.5 times.^10, 20^ The association between delirium and mortality may be attributed to direct mechanisms such as neuroinflammation, neurotransmitter imbalance, and cerebral metabolic disturbances, all of which can lead to long-term neuronal damage. Indirectly, delirium contributes to increased mortality through complications like aspiration pneumonia, pressure ulcers, and the use of physical restraints. Prolonged hospital and intensive care unit stays due to delirium further elevate the risk of hospital-acquired complications.^21^

The elevated mortality observed in patients with delirium can also be explained by higher APACHE II and SOFA scores, which are predictive models for multiple physiological parameters and organ systems. As a multifactorial syndrome, delirium is inherently linked to higher scores on these assessments, reflecting an increased risk of adverse outcomes. Multicentre studies and meta-analyses have demonstrated a correlation between elevated APACHE II scores and increased delirium risk.^22, 23^ In our study, we observed a higher frequency of delirium with increasing APACHE II scores. The mean APACHE II score was 5 points higher in patients with delirium than in those without delirium. In a study conducted by Salluh et al.^24^, the median SOFA score was 4 in patients with delirium and 3 in the non-delirium group. Similarly, in our study, the mean SOFA score of the patients with and without delirium was 4.3 and 2.9, respectively. In our study, the expected mortality rate for patients with delirium based on APACHE II scores was approximately 25%, whereas the expected mortality rate based on SOFA scores was 10%, which is consistent with the findings in the literature. The elevated mortality rate in the delirium group is consistent with these expectations.^25, 26^ These results emphasize the critical importance of heightened vigilance for delirium in patients with higher APACHE II and SOFA scores, which are significant predictors of poor clinical outcomes in this population.

Advanced age, particularly when accompanied by visual and hearing impairments, is recognized as a significant risk factor for delirium.^27, 28^ Individuals with vision or hearing loss are up to three times more likely to develop delirium. However, the use of assistive devices for these impairments in hospitalized elderly patients can prevent delirium and reduce its duration.^2, 28^ In this study, we also found that advanced age and the presence of visual or hearing impairments were risk factors for delirium. We also determined that there was a remarkable relationship between the development of delirium and education level and that the lowest incidence was among university graduates. These findings suggest that susceptibility to delirium increases as cognitive function declines.

Bellelli et al.^29^ showed the effect of interventional procedures such as nasogastric tubes, central venous catheters, and urinary catheters on delirium. Additionally, in a multicenter delirium epidemiology in critical care-DECCA study including 975 patients, a relationship was found between central venous catheters, arterial catheters, urinary catheters, and delirium.^26^ In agreement with the literature, we found similar results regarding the relationship between nasogastric and wound drain catheter use and delirium. Moreover, a noteworthy finding of our study was the association between the number of aspirations performed during the day and the risk of delirium. However, we did not observe a significant association between the use of urinary catheters and delirium. This discrepancy could be attributed to the high frequency of urinary catheter use in our patients.

Urinary retention and constipation are recognized risk factors for delirium. Smonig et al.^30^ demonstrated an increased incidence of delirium in patients in whom defecation was absent for more than 5 days. In line with these findings, our study revealed a similar trend, indicating a higher incidence of delirium in patients who did not defecate for more than 3 days and had decreased urine output.

Infection and sepsis are significant risk factors for delirium. The pathophysiological mechanisms involved in these conditions include neuroinflammation and microglial activation resulting from infection as well as impaired cerebral perfusion and neurotransmitter imbalance. These factors are believed to contribute to delirium.^31^ In the present study, we found a higher prevalence of microbiological growth in the blood, urine, and endotracheal aspirate cultures of patients with delirium, supporting these hypotheses.

Malnutrition is a risk factor for delirium, particularly in the elderly population of ICU patients, and enteral nutrition is effective in reducing delirium by preventing malnutrition.^32^ Few studies have compared oral and enteral nutrition in the existing literature. In the present study, we discovered a protective effect of oral nutrition against the development of delirium. Specifically, compared with oral enteral nutrition, enteral nutrition with a feeding tube increased the risk of delirium by 2.5-fold.

In our study, we noticed a 2.9-fold increase in the likelihood of delirium by 2.9 times among patients who received an infusion for sedation. Zaal et al.^33^ found that intravenous infusion increased the risk of delirium by 4% on the subsequent day in ICU patients. However, this effect was not observed with the bolus. Consistent with these findings, our study revealed that administering sedatives as a bolus had no impact on delirium emergence, whereas infusion increased the risk of delirium by 3.4 times.

Corticosteroids can disrupt behavioral and cognitive functions by affecting serotonergic neurotransmitters within the intracellular space. Our study also found that steroid use increased the risk of developing delirium.

We observed a significant 2.3-fold increase in delirium among patients who received albumin replacement. However, it is uncertain whether this risk is caused directly by albumin administration or hypoalbuminemia. Hypoalbuminemia can potentially contribute to delirium through two mechanisms. Low albumin levels may have adverse effects on hemodynamics by reducing intravascular oncotic pressure. Low oncotic pressure may compromise hemodynamic stability and predispose patients to delirium. Second, hypoalbuminemia may influence the pharmacokinetics of drugs that affect cognitive function. The drugs used in clinical practice rely on albumin for transport and distribution. Hypoalbuminemia alters the availability and concentration of these drugs, potentially influencing their effectiveness and adverse effects. Such disruptions in drug metabolism and distribution may have implications for cognitive function and, consequently, contribute to the emergence of delirium.

In this study, we did not find any significant association between blood glucose levels, electrolyte disorders, and the emergence of delirium. Although several studies have suggested a connection between metabolic and serum electrolyte disorders and delirium, no consensus has been reached on this matter.^10, 12^

Hypoxic ischemic encephalopathy can cause severe neuronal and cortical damage, particularly if left untreated, and may present as hypoactive and mixed delirium during the early stages of cerebral hypoxia and hypercarbia. Therefore, it is important to identify hypoxia-induced delirium in patients. In our study, there was no association between delirium and hyperoxia, whereas hypoxia and hypercarbia were associated with an increased risk of delirium.

To prevent the onset of delirium, it is crucial to address the factors that limit patient movement and prioritize mobilization through regular physical therapy with at least three sessions per day.^10^ Although the incidence of delirium increased among patients with movement restraints in our study, the results were not statistically significant. However, mobilization had a protective effect against the development of delirium.

In the pain agitation/sedation, delirium, immobility, and sleep disruption guidelines, the use of bright light should be minimized because of its potential impact on delirium.^16^ Our findings support this recommendation, as we demonstrated that decreased sleep quality is associated with a 4.6-fold increased risk of delirium. The lighting systems in these units may play a role in affecting sleep quality, thereby contributing to the development of delirium.

High pain scores are a risk factor for the development of delirium, and the use of analgesics is recommended.^16^ However, in our study, no relationship was found between the average pain score and the development of delirium. This is attributed to analgesic practices that did not allow VAS scores to exceed three points.

In a previous study, patients with delirium were reported to have a hospitalization duration of approximately 11 days before ICU admission.^34^ However, in our study, the duration of hospitalization before ICU admission for patients with delirium was shorter (5.9 days. Despite this discrepancy, our study demonstrated that the frequency of delirium increased with the duration of hospitalization before ICU admission.

Ely et al.^18^ reported a higher incidence of delirium in patients receiving mechanical ventilation. Similarly, 53.3% of patients on mechanical ventilators developed delirium. In addition, intubation and oxygen requirement were significant risk factors. However, continuous positive airway pressure, tracheostomy, and reintubation were not found to be associated with delirium. It is possible that the lack of a significant association between these latter factors could be attributed to the relatively small number of patients in these subgroups.

Delirium has multiple causes, including a history of hypertension, vasopressor use, and hemodynamic instability, which increase the risk. In this study, we also found that hemodynamic instability, hypertension history, and vasopressor use were associated with delirium in intensive care patients.

It is stated that delirium in the ICU usually begins on the second day of hospitalization and lasts for an average of 3 days.^34^ Similarly, delirium developed on the second day after admission, and the average duration of delirium was 5.8 days in our study.

In the pharmacologic management of delirium, various agents, including antipsychotics-particularly haloperidol-alpha-2 agonists, such as dexmedetomidine, antidepressants, and acetylcholinesterase inhibitors, have been explored in the literature.^16^ In our study, dexmetatomidine and haloperidol were the most frequently used treatments, yet no significant difference was observed in terms of patient outcomes like discharge rates or mortality. Combinations of these drugs were used in a small subset of patients, although the lack of statistical significance in outcomes suggests that a larger sample size is needed to explore potential additive or synergistic effects. Current guidelines favor dexmedetomidine for its sedative properties without respiratory depression, making it a preferable option in certain ICU settings.^13, 14^ However, the observed variability in treatment success underscores the need for individualized approaches based on patient condition.

### Study Limitations

A primary limitation of this study was the twice-daily delirium assessment schedule, which, while providing consistent monitoring, may have led to an underestimation of the true incidence of transient delirium, especially in patients with fluctuating cognitive states. More frequent assessments, such as every four to six hours, may provide a more accurate representation of the prevalence of delirium in critical care settings. If patients had been evaluated more frequently throughout the study, the recorded incidence of delirium might have been higher. Additionally, in this study, the cognitive status of patients after discharge from the ICU was not assessed. Our assessment did not include factors such as the extent of care delivered by healthcare providers and patient relatives and the potential increase in hospital costs.

## Conclusion

Nearly half of the delirium cases in this study were hypoactive and often under-recognized. Timely identification of patients is essential for improving outcomes. The study also identified several risk factors, including advanced age, sensory impairments, educational status, hypertension, heart disease, hemodynamic instability, catheter use, infection, sedative infusions, constipation, albumin and steroid use, enteral nutrition, mechanical ventilation, prolonged intensive care unit stays, and elevated SOFA and APACHE II scores. Addressing these factors early can help reduce the incidence of delirium in the ICU.

## Ethics

**Ethics Committee Approval:** This prospective observational study was approved by the Gazi University Faculty of Medicine Clinical Research Ethics Committee (decision no.: 588, date: 10.9.2018).

**Informed Consent:** Written informed consent was obtained from each patient.

## Figures and Tables

**Table 1. Indications for Admission to the Anaesthesiology, General Surgery, and Neurology ICUs:** 

**Indications for admission**	**Anaesthesiology** **n (%)**	**General Surgery** **n (%)**	**Neurology** **n (%)**	**Total** **n (%)**
Postoperative	32 (38.6)	51 (61.4)	0 (0)	83 (39.2)
Cerebrovascular incident	5 (15.6)	1 (3.1)	26 (81.3)	32 (15.1)
Respiratory failure	19 (82.6)	2 (8.7)	2 (8.7)	23 (10.8)
Posttrauma/accident	23 (100)	0 (0)	0 (0)	23 (10.8)
Acute abdomen	2 (12.5)	12 (75.0)	2 (12.5)	16 (7.5)
Multiorgan dysfunction	5 (35.7)	7 (50)	2 (14.3)	14 (6.6)
Intoxication	7 (100)	0 (0)	0 (0)	7 (3.3)
Kidney failure	3 (75.1)	1 (25)	0 (0)	4 (1.9)
Sepsis	3 (75)	1 (25)	0 (0)	4 (1.9)
Post CPR	2 (66.7)	1 (33.3)	0 (0)	3 (1.4)
Cardiac instability	1 (50)	1 (50)	0 (0)	2 (0.9)
Liver failure	0 (0)	1(100)	0 (0)	1 (0.5)
Total	102 (48.1)	78 (36.8)	32 (15.1)	212

ICU, intensive care unit; CPR, cardiopulmonary resuscitation.

**Table 2. Non-modifiable Risk Factors and Demographic Characteristics of Delirium:** 

**Properties**	**Deliriuma**	**No Deliriuma**	**Total**	***P***
**ICU n (%)**				
Anaesthesia ICU General surgery ICU Neurology ICU	36 (52.2) 21 (30.4) 12 (17.4)	66 (46.2) 57 (39.9) 20 (14.0)	102 (48.1) 78 (36.8) 32 (15.1)	0.4^1^
**Gender n (%)**				
Male Female	34 (49.3) 35 (50.7)	71 (49.7) 72 (50.3)	105 (49.5) 107 (50.5)	0.95^1^
Age (mean ± SD)	67.0±16.5	52.4±19.4	57.5±19.7	<0.001^2^
Body mass index (mean ± SD)	25.9±6.5	26.0±6.8	25.9±6.7	0.99^2^
**Education level (%)**				
Illiterate Primary school graduate Secondary school graduate High school graduate University graduate	9 (13.0) 31 (44.9) 13 (18.8) 8 (11.6) 8 (11.6)	9 (6.3) 57 (39.9) 15 (10.5) 30 (21.0) 32 (22.4)	18 (8.5) 88 (41.5) 28 (13.2) 38 (17.9) 40 (18.9)	0.03^1,*^
**Comorbidities, n (%)**				
Visual impairment Hearing impairment Smoking Ex-smoker Alcohol Hypertension Epilepsy Lung disease Heart disease Liver disease Diabetes Malignancy Trauma history	41 (59.4) 23 (33.3) 20 (29.0) 23 (33.3) 6 (8.7) 42 (60.9) 3 (4.3) 14 (20.3) 18 (26.1) 3 (4.3) 18 (26.1) 27 (39.1) 7 (10.1)	39 (27.3) 18 (12.6) 31 (21.7) 31 (21.7) 10 (7.0) 54 (37.8) 2 (1.4) 16 (11.2) 19 (13.3) 5 (3.59) 32 (22.4) 53 (37.1) 22 (15.4)	80 (37.7) 41 (19.3) 51 (24.1) 54 (25.5) 16 (7.5) 96 (45.3) 5 (2.4) 30 (14.2) 37 (17.5) 8 (3.8) 50 (23.6) 80 (37.3) 29 (13.7)	<0.001^1,*^ <0.001^1,*^ 0.24 0.06 0.6 0.002^1,*^ 0.18 0.07 0.02^1,*^ 0.76 0.55 0.77 0.29
**Transferring wards n (%)**				
Emergency room Other Postoperative External center	37 (53.6) 23 (33.4) 7 (10.1) 2 (2.9)	65 (45.5) 39 (27.2) 34 (23.8) 5 (3.5)	102 (48.1) 62 (29.3) 41 (19.3) 7 (3.3)	0.21^1^
SOFA score (mean ± SD)	4.3±2.5	2.9±2.7	3.4±2.8	<0.001^2,*^
APACHE II score (mean ± SD)	16.9±5.4	11.9±5.0	13.6±5.7	<0.001^2,*^
Pre-ICU hospital length of stay (mean ± SD)	5.9±12.5	4.6±17.1	5.0±15.7	0.8
Mortality	17 (24.6)	7 (4.9)	24 (11.3)	<0.001^1,*^

^1^Pearson chi-square test, ^2^Mann-Whitney U test,^ a^Column percentage, ^*^P < 0.05

ICU, intensive care unit; SD, standard deviation; SOFA, sequential organ failure assessment; APACHE, acute physiology and chronic health evaluation.

**Table 3. Modifiable Risk Factors for Delirium Development:** 

	**Delirium^a ^n (%)**	**No Delirium^a ^n (%)**	**Univariate OR (95% CI Lower Bound-Upper Bound)**	***P^1^***
**Interventions**				
Nasogastric catheter Urinary catheter Wound drain Tracheal aspiration Central catheter Arterial catheter Pneumatic device	32 (46.4) 65 (94.2) 14 (20.3) 30 (43.5) 23 (33.3) 13 (18.8) 5 (7.2)	41 (28.7) 128 (89.5) 52 (36.4) 25 (17.5) 33 (23.1) 17 (11.9) 14 (9.8)	2.15 (1.18-3.90) 0.25 (0.12-0.51) 2.89 (1.39-6.02) 3.63 (1.91-6.90) 1.66 (0.88-3.14) 0.68 (0.27-1.69) 2.39 (0.74- 7.65)	0.01* 0.26 0.01* <0.001* 0.11 0.17 0.54
**Nutrition**				
Enteral nutrition Oral enteral nutrition Parenteral nutrition	14 (20.3) 11 (15.9) 12 (17.4)	13 (9.1) 59 (41.3) 23 (16.1)	2.54 (1.12-5.76) 0.27 (0.13-0.55) 0.91 (0.42-1.9)	0.02* <0.001* 0.8
**Ventilation**				
Mechanical ventilation Oxygen requirement Hypoxia CPAP Intubation	24 (34.8) 55 (79.7) 3 (4.3) 3 (4.3) 16 (23.2)	21 (14.7) 82 (57.3) 5 (3.5) 5 (3.5) 18 (12.6)	3.09 (1.57-6.10) 2.92 (1.49-5.73) 2.05 (1.03-4.08) 0.7 (0.18-3.43) 2.09 (0.99-4.42)	0.001* 0.001* 0.76 0.04* 0.05
**Transfusion** ES transfusion FFP transfusion Platelet transfusion	14 (20.3) 10 (14.5) 9 (13.0) 3 (4.3)	40 (28.0) 31 (21.7) 21 (14.7) 2 (1.4)	1.71 (0.36-8.00) 0.64 (0.17-2.36) 1.21 (0.32-4.61) 0.31 (0.05-1.91)	0.22 0.21 0.74 0.18
**Drugs**				
Midazolam infusion Midazolam bolus Propofol infusion Vasopressor Insulin Albumin Steroid	9 (13.0) 7 (10.1) 9 (13.0) 9 (13.0) 14 (20.3) 16 (23.2) 18 (52.6)	6 (4.2) 7 (4.9) 7 (4.9) 8 (5.6) 20 (14.1) 16 (11.2) 20 (47.4)	3.4 (1.16-10.05) 2.19 (0.7-6.52) 2.91 (1.03-8.19) 2.53 (0.93-6.87) 0.64 (0.3-1.3) 2.39 (1.11-5.14) 2.17 (1.06-4.40)	0.02* 0.15 0.04* 0.06 0.25 0.02* 0.03*
**Urine output**				
Normal Anuric/Oligouric	39 (56.5) 30 (43.5)	103 (72.0) 40 (28.0)	0.50 (0.27-0.92) 1.56 (1.06-2.28)	0.02*
**Defecation frequency**				
<3 days >3 days	20 (15.4) 49 (71.0)	22 (29.0) 121 (84.6)	0.44 (0.22-0.88) 1.65 (1.11-2.45)	0.02*
**Hemodynamic instability**				
Hypotension Hypertension	11 (15.9) 18 (26.1)	10 (7.0) 19 (13.3)	2.5 (1.01-6.26) 2.20 (1.11-4.74)	0.04* 0.02*
**Blood glucose level**				
Hypoglycemics Hyperglycemic	4 (5.8) 15 (21.7)	4 (2.8) 21 (14.8)	0.59 (0.28-1.25) 1.4 (0.30-6.50)	0.22
**Growth in culture** Blood culture (+) Urine culture (+) ETA culture (+) Wound site culture (+) Sepsis	39 (56.5) 18 (26.1) 15 (21.7) 15 (21.7) 12 (17.4) 13 (18.8)	44 (30.8) 20 (14.0) 14 (9.8) 7 (4.9) 20 (14.0) 7 (4.9)	2.92 (1.61-5.29) 2.17 (1.06-4.40) 2.56 (1.15-5.66) 5.39 (2.08-13.96) 1.29 (0.59-2.82) 4.51 (1.71-11.89)	<0.001* 0.03* 0.01* <0.00* 0.516 0.001*
History of previous surgery	26 (37.7)	84 (58.7)	0.42 (0.23-0.76)	0.004*
History of previous anaesthesia	23 (33.3)	78 (54.5)	0.41 (0.22-0.75)	0.004*

^1^Pearson chi-square test,^ a^column percentage, ^*^P < 0.05

CPAP, continuous positive airway pressure; ES, erythrocyte suspension; FFP, fresh frozen plasma; ETA, endotracheal aspirate; OR, odds ratio; CI, confidence interval.
